# Automatic Classification of Cancer Pathology Reports: A Systematic Review

**DOI:** 10.1016/j.jpi.2022.100003

**Published:** 2022-01-20

**Authors:** Thiago Santos, Amara Tariq, Judy Wawira Gichoya, Hari Trivedi, Imon Banerjee

**Affiliations:** a Department of Computer Science, Emory University, Atlanta, GA, USA; b Department of Biomedical Informatics, Emory School of Medicine, Atlanta, GA, USA; c Department of Radiology, Emory School of Medicine, Atlanta, GA, USA; d Department of Radiology, Mayo Clinic, Phoenix, AZ, USA; e Department of Computer Engineering, Arizona State University, AZ, USA

## Abstract

Pathology reports primarily consist of unstructured free text and thus the clinical information contained in the reports is not trivial to access or query. Multiple natural language processing (NLP) techniques have been proposed to automate the coding of pathology reports via text classification. In this systematic review, we follow the guidelines proposed by the Preferred Reporting Items for Systematic Reviews and Meta-Analyses (PRISMA; Page et al., 2020: *BMJ*.) to identify the NLP systems for classifying pathology reports published between the years of 2010 and 2021. Based on our search criteria, a total of 3445 records were retrieved, and 25 articles met the final review criteria. We benchmarked the systems based on methodology, complexity of the prediction task and core types of NLP models: i) Rule-based and Intelligent systems, ii) statistical machine learning, and iii) deep learning. While certain tasks are well addressed by these models, many others have limitations and remain as open challenges, such as, extraction of many cancer characteristics (size, shape, type of cancer, others) from pathology reports. We investigated the final set of papers (25) and addressed their potential as well as their limitations. We hope that this systematic review helps researchers prioritize the development of innovated approaches to tackle the current limitations and help the advancement of cancer research.

## Introduction

Although the cancer death rate has dropped 2.2% since 2016, it remains the second leading cause of death in the United States, and it is estimated that 1.9 million Americans will be diagnosed with cancer in 2021.^2^^,^^3^ In the majority of the cases, a biopsy is performed, and the diagnosis is made via histopathological analysis. During this process, pathologists record highly descriptive and specific observations of cells, organs, and tissue specimens in unstructured and/or semi-structured pathology reports. These reports contain immense quantities of relevant information, which is critical to advance cancer research in fields like treatment selection, case identification, prognostication, surveillance, clinical trial screening, risk stratification, retrospective study, and many others.^4^, ^5^, ^6^, ^7^ One essential challenge when retrieving these important descriptive observations from pathology reports is that a large portion of the diagnosis is encoded in an unstructured, free-text format. State or national cancer registries who track thousands or millions of patients typically must extract this relevant information by using human experts to code information to a normalized and structured form. This manual processing of information is time-consuming, costly, error prone, and also imposes inherent limitations on the volume and types of information that can be extracted.

To address these limitations and automate the information extraction (IE) process on pathology reports, NLP has recently received much attention from the cancer research communities.^8^, ^9^, ^10^ While a surge of research has been published, only a few review/survey articles are available in this area - with the latest one published in 2016,^11^ which mainly focuses on word/phrase matching, probabilistic statistical machine learning, and rule-based systems. Since then, the IE field in pathology has moved rapidly towards deep learning^12^, ^13^, ^14^ and to our knowledge, no comprehensive review article exists to cover the recent deep learning-based IE systems and benchmark them against existing rule-based systems. Given the breadth of the pathology IE field, there is a need to properly summarize these works and the current status of the field to provide a steppingstone for future advancements.

In order to fill the gap, we performed a systematic review following the standard PRISMA guidelines^1^ to describe the current literature in the area of IE for pathology reports - primarily focusing on classification of diagnosis labels and information extraction from cancer pathology reports. We assess how these systems have been assisting in developing clinical decisions by extracting relevant information from pathology reports and how they have improved the quality of data creation. We benchmark the systems based on the type of AI model, architecture, intelligent techniques, and performance measures. The remainder of the paper is organized as follows: Section 2 - detailed methodology; Section 3 - main findings; Section 4 - conclusion, including main contributions, limitations, and future research directions in the field.

## Methodology

This systematic review followed the PRISMA guidelines^1^ to identify, select, and critically appraise all relevant research in natural language processing systems that automatically classify and/or extract information from cancer pathology reports.

**Figure 1:**
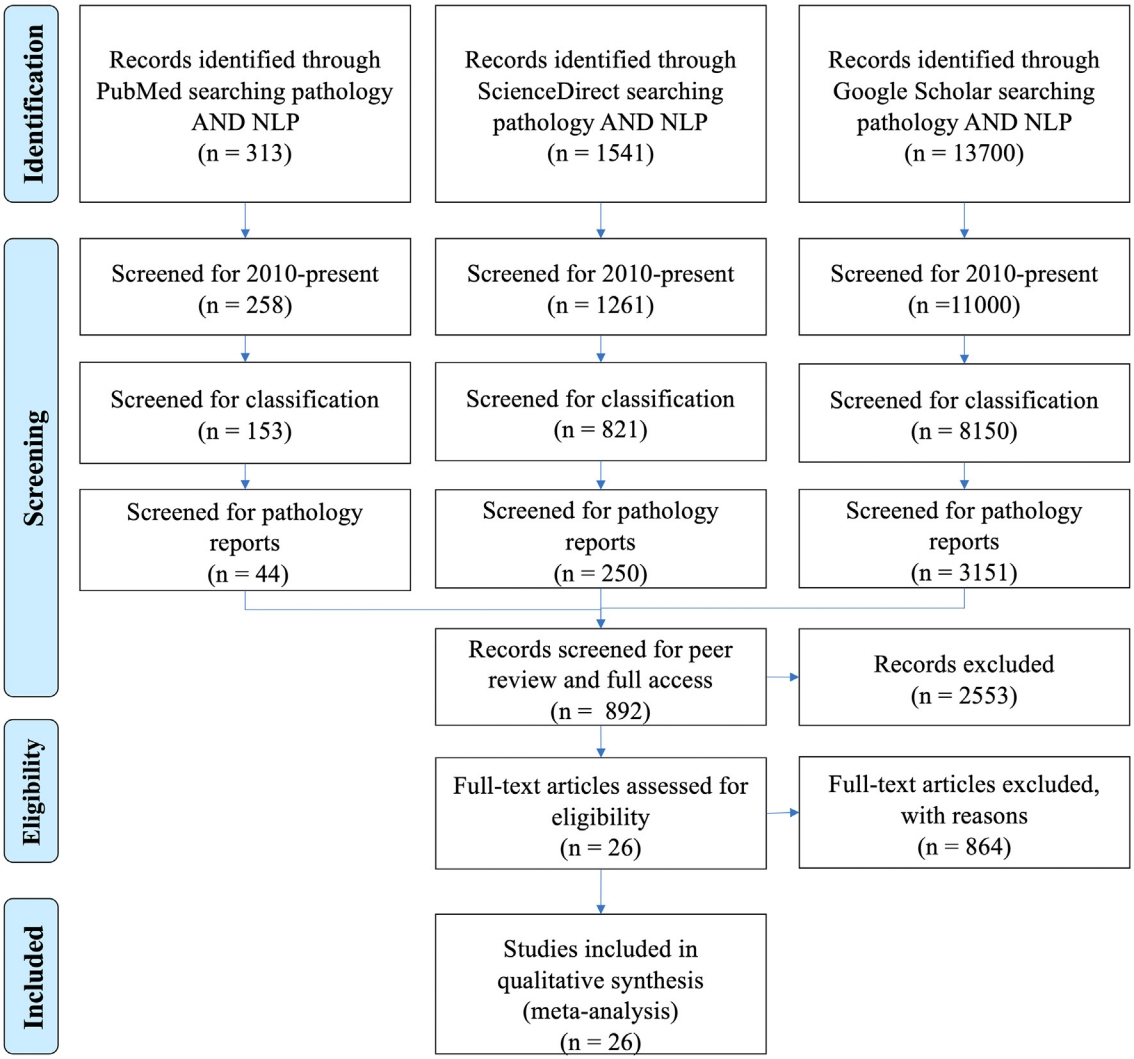
Flow diagram of the search and inclusion process in the study. This study was reported according to the Preferred Reporting Items for Systematic Reviews and Meta-Analyses guidelines.^1^

In this systematic literature review, we retrieved articles from three popular literature search engines: PubMed, Science Direct, and Google Scholar. Afterwards, three reviewers with varying experience level (TS (MS - Phd student), AT (Ph.D., Postdoc), and IB (Ph.D, Assis. Prof.)) screened all retrieved papers for relevance during screening phases, and the reported results were based on the final set of selected papers. The overall search strategy is illustrated on Fig. 1.

The first step was to identify and retrieve papers based on the following keywords: pathology **AND** cancer **AND** natural language processing. Secondly, we excluded papers published before 2010 to focus on the recent literature, yielding 3445 unique papers from three search engines. The primary exclusion criteria were: (i) articles that were not peer reviewed, such as those in arXiv, (ii) articles that lack methodological details, (iii) articles not written in English, and (iv) studies that did not primarily focus on NLP.

Next, we refined our selected papers to contain only articles related to text classification and information extraction for cancer. In addition, we excluded articles which did not use pathology reports as the core input dataset. These key steps were important to retrieve the relevant papers related to the focus of the review.

**Figure 2:**
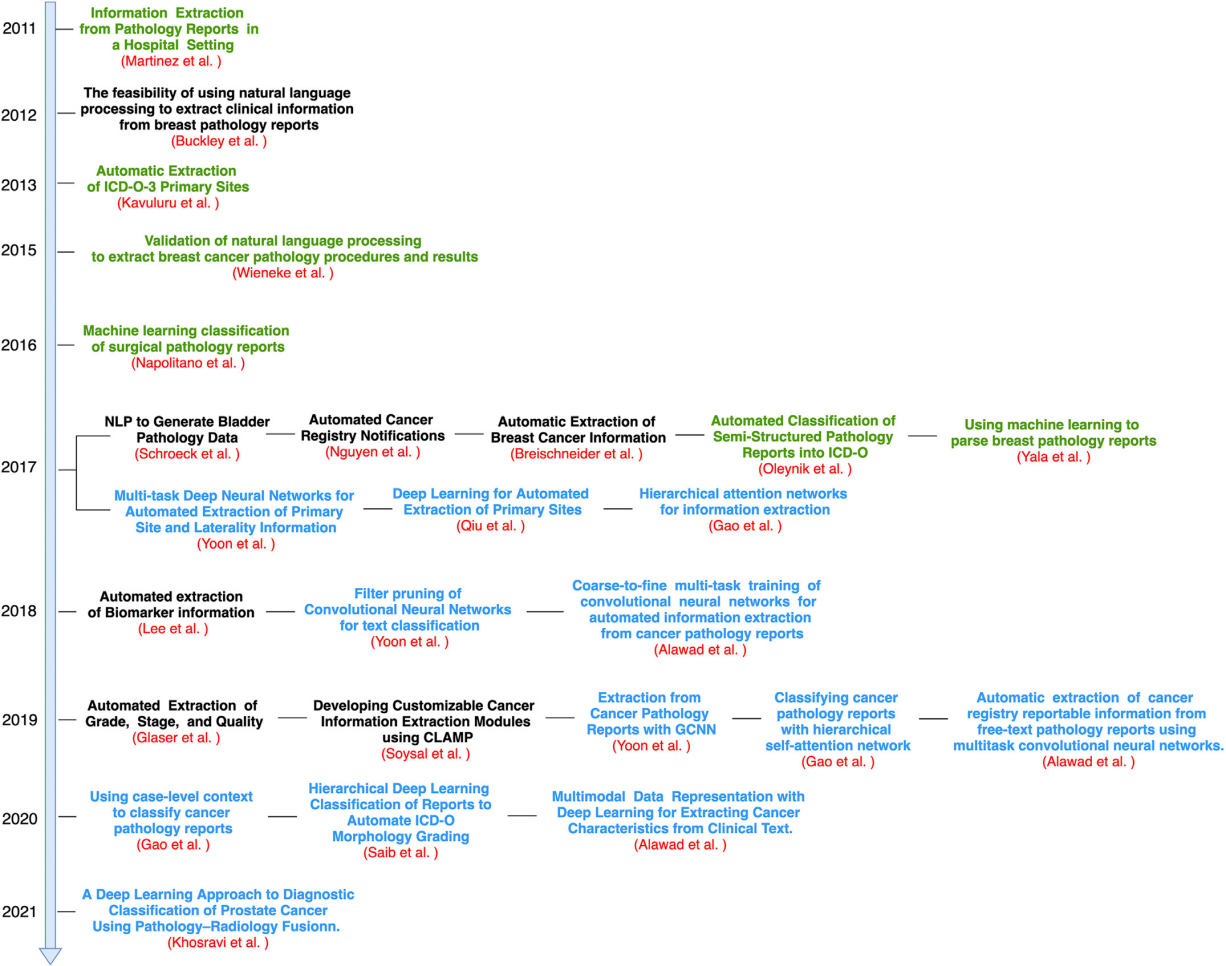
A temporal illustration of the reviewed papers included in this systematic review according to the publication date. Each color indicates different aspects of the review: (i) black indicates a rule-based and intelligent system, (ii) green indicates a statistical machine learning model, and (iii) blue indicates a deep learning model

Lastly, we reviewed the papers methodology and model architecture. Many papers have primarily focused on the clinical aspects but did not provide sufficient details about the applied NLP methods. Therefore, we excluded the papers which failed to describe the core benchmarking criteria of our study - IE problem, the architecture of the model, the datasets, or the evaluation metrics. Furthermore, we also excluded papers which only used a commercial NLP software as their main model (e.g., DeepPhe^15^). After these steps, 25 studies remained matching the study criteria. Papers were categorized into types of statistical machine learning systems: (i) rule-based and intelligent systems, (ii) statistical machine learning, (iii) deep learning. Figure 2 represents a temporal publication timeline of the papers included in this literature review in ascending order of publication date along with its category.

## Results

From the literature search, 25 papers were selected when applying the inclusion and exclusion criteria (see Section 2). The majority of the selected papers aimed to develop NLP models to extract common cancer characteristics (grade, stage, laterality, etc.) from pathology reports by formulating the information extraction task as a classification problem. Other objectives included prediction of International Classification of Diseases (ICD) codes from cancer pathology reports. Table 1 summarizes the studies and in the following section, we discussed the results in the following categories: (i) rule-based and intelligent systems, (ii) statistical machine learning-based, and (iii) deep learning-based.

**Table 1:** Benchmarking of the natural language processing systems for pathology reports - prediction task, data and evaluation. Listed in ascending ordered according to the year of publication 2021–2010.

Citation	Year	Methodology	Prediction Task	Data	Evaluation
Khosravi et al.^41^	2021	Deep learning	Classify cancer vs. benign and high vs. low-risk of prostate disease	Local urology center database of 400 prostate cancer MRI images and pathology re-ports	AUCs of 0.89 and 0.78 for classification of cancer vs benign and high vs low risk, respectively
Gao et al.^50^	2020	Hierarchical deep learning	Six cancer classification tasks: site, subsite, laterality , histology, behavior, and grade	546, 806 cancer (all types) pathology re-ports obtained from the SEER cancer registry program	F1 Micro of 0.92, 0.64, 0.92, 0.8, 0.98, and 0.82 for site, subsite, laterality, histology, behavior, and grade, respectively
Saib et al.^51^	2020	Hierarchical deep learning	Classify 9 ICD-O morphology grading	1813 breast cancer pathology reports obtained from a local center database	F1 Micro of 0.91 and F1 Macro of 0.69 for classification of 9 ICD-O codes
Alawad et al.^43^	2020	Deep learning	Two cancer classification tasks: subsite with 317 labels and histology with 556 labels	878,864 cancer (all types) pathology reports obtained from the SEER cancer registry program	F1 Micro of 0.68 for subsite; F1 Micro of 0.79 for histology
Glaser et al.^22^	2019	Rule-Based	Extract stage, grade, and presence of muscularis propria	3,042 Transurethral Resection of the Bladder Tumor (TURBT) reports obtained from a local database	Accuracy of 82%, 88% , and 100% for extracting stage, specimens and grade, respectively
Soysal et al.^23^	2019	Rule-based	Extract cancer-related information in pathology reports (e.g., tumor size, tumor stage, specimen, biomarkers, and others)	400 cancer (all types) pathology reports obtained from a local center database	F1 average performance ranging from 0.87 to 0.99 for extracting cancer information
Yoon et al.^40^	2019	Multi-task deep learning	Four cancer classification tasks: subsite, laterality, behavior, and histological grade	942 unstructured cancer (all types) pathology reports obtained from the SEER cancer registry program	F1 Micro of 0.98, 0.98, 0.99, and 0.97, for Site, Laterality, Behavior, and Grade, respectively F1 average of 0.98
Gao et al.^49^	2019	Hierarchical deep learning	Five cancer classification tasks: site, laterality, behavior, histology, and grade.	374,899 cancer (all types) pathology reports obtained from the SEER cancer registry program	Accuracy of 0.9, 0.89, 0.96, 0.76, 0.71 for site, laterality, behavior, histology, and grade, respectively
Alawad et al.^56^	2019	Multi-task deep learning	Five cancer classification tasks: site, laterality, behavior, histology, and grade.	95,231 (all types) pathology reports obtained from the SEER cancer registry program	F1 Micro of of 0.94, 0.82, 0.95, 0.65, and 0.76 for site, laterality, grade, and behavior, re- spectively
Lee et al.^21^	2018	Rule-based	Extract tissue slide identifier, biomarker names, and test result identifier	867 bladder tumor (TURBTs) Pathology Reports obtained from a local center database	F1 score of 0.99, 0.97, and 0.96 for extracting tissue slide identifier, biomarker names, and test result, respectively Accuracy of 0.88
Yoon et al.^39^	2018	Deep learning	Classify 12 ICD codes	942 pathology reports (breast and lung) obtained from a local center database	F1 Micro of 0.78 for classification of 12 ICD-O codes
Alawad et al.^55^	2018	Multi-task deep learning	Three cancer classification tasks: site, laterality, and histology	942 site, 642 histology, and 815 laterality pathology reports obtained from a local center database	F1 Micro of 0.77, 0.79, and for primary site, grade, and laterality, respectively
Qiu et al.^38^	2017	Deep learning	Classify 12 ICD codes	942 pathology reports (breast and lung) obtained from a local center database	F1 Micro of 0.72 for classification of 12 ICD-O codes
Gao et al.^47^	2017	Hierarchical deep learning	Two cancer classification tasks: primary site, and grade	942 cancer (all types) pathology reports obtained from the SEER cancer registry program	F1 Micro score of 0.8 and 0.91 for primary site and histological grade, respectively
Schroeck et al.^4^	2017	Rule-based	Extract histology, invasion , grade, carcinoma, and presence of muscularis propria	600 bladder pathology reports obtained from a local center database	Accuracy ranged from 0.83 to 0.96 for extracting histology, invasion, grade, carcinoma, and muscularis.
Nguyen et al.^19^	2017	Rule-based	Identify cancer notifiable patients	45.3 million pathology HL7 messages	Sensitivity of 0.96 and specificity of 0.96
Breischneider et al.^20^	2017	Rule-Based	Extraction of size, grading, hormone, and lymph nodes	8,766 breast cancer reports obtained from a local center database	Accuracy of 0.41, 0.77, 0.86, and 0.78 for extracting size, grading, hormone, and lymph nodes, respectively
Oleynik et al.^32^	2017	Statistical Machine Learning	Classify ICD codes	94,000 pathology reports obtained from a local center database	F1 of 0.82 and 0.73 for classifying ICD codes from topography and morphology classes.
Yala et al.^33^	2017	Statistical Machine Learning	IE of tumor characteristics	91,000 breast pathology reports	F1 score of 0.92
Napolitano et al. ^57^	2016	Statistical Machine Learning	Classify pathology reports and chunk recognition	798 surgical pathology reports obtained from a local center database	Accuracy of 0.994
Wieneke et al.^31^	2015	Statistical Machine Learning	Three classification tasks: procedure, laterality, and result	3234 pathology reports	F1 Micro score of 0.8, 0.92, and 0.5 for procedure, laterality, and result, respectively
Kavuluru et al.^30^	2013	Statistical Machine Learning	Classify ICD-O-3 codes	56,000 pathology reports obtained from a local center database	F1 Micro of 0.9 and F1 Macro of 0.71 for classification of ICD- O-3 codes F1 score of 0.93
Buckley et al.^16^	2012	Rule-based	IE of cancer characteristics	76,333 breast pathology reports obtained from a local center database	Specificity of 0.96
Martinez et al.^29^	2011	Statistical Machine Learning	IE of cancer characteristics	217 clinical records obtained from a local center database	F1 of 0.58 and 0.7 for Tumor Site and Nodes Examined, respectively

### Rule-based and Intelligent Systems

Rule-based natural language processing systems rely on expert-developed rules to understand free-flowing text and extract information such as named entities, interesting concepts, and relationships between entities of interest. In the context of pathology reports, rule-based NLP was a popular choice for processing pathology reports through 2019 at which point both rule-based and statistical machine learning-based systems were replaced in favor of deep learning although some rule-based systems continued to be developed through 2019. In our review, we included nine rule-based systems.

Earlier studies have focused mostly on applying rule-based NLP techniques for IE on pathology reports with varying results.^4^^,^^16^, ^17^, ^18^, ^19^, ^20^, ^21^, ^22^, ^23^ Buckley et al.^16^ elaborated a combination of rules for extracting cancer entities (invasive ductal cancer, invasive lobular cancer, ductal carcinoma in situ, atypical ductal hyperplasia, lobular carcinoma in situ, and usual ductal hyperplasia and its variations, from pathology reports. Nguyen et al.^19^ designed a set of rules in combination with Medtex^24^ and SNOMED^25^ concepts to identify if malignancy is presented in the pathology report. Glaser et al.^22^ leveraged the combination of rule-based techniques, heuristics, and regular expressions to extract the cancer stage - low grade, high grade, and presence of muscularis propria from bladder cancer pathology reports. These rule-based systems require domain expertise and large amounts of manual effort in designing specific and carefully crafted rules for IE. Furthermore, the design of such rules brittle and susceptible to small changes in language, distribution of pathology, or redefinition of pathology terms, and is therefore unsustainable in long-term.

### Statistical Machine Learning

An alternative approach to manual rule engineering is to employ statistical machine learning techniques like Support Vector Machines, Logistic Regression, and Random Forest classifiers.^26^, ^27^, ^28^ Instead of using manually curated rules, these approaches use a hidden vector space representation of the textual reports to learn patterns in the data to automate extraction of relevant terms/information from pathology reports.^29^, ^30^, ^31^, ^32^, ^33^ However, such systems need supervision in the form of labeled data.

To automate the extraction of ICD codes from cancer pathology reports, Kavuluru et al.^30^ used Unified Medical Language System (UMLS)^34^ entities to map the text and utilized Concept Unique Identifiers (CUIs) from UMLS codes as feature inputs to a support vector machine model for classification. To automatically convert free-text pathology reports into structured data, Yala et al^33^ presented a boosting classifier using weak learners to identify tumor features. Their primary contribution was the establishment of a database of pathology report features that could be utilized to identify patient groups with certain characteristics. The main drawback of these methods is that they are not able to capture either the semantic or syntax information from the text when making a prediction. In other words, they are not able to create complex sentence representations since often they only consider the presence or frequency of word occurrences, irrespective of their ordering in a sentence which could have significant impact in actual meaning of the text.

### Deep Learning

Deep learning algorithms have improved upon the limitations of traditional statistical machine learning models and demonstrated superior performance in many NLP tasks, including *Text Classification* and *Information Extraction*. These improvements are strongly related to the deeper network designs and their ability to create complex sentence representations known as word embeddings. Distributed representation of words involves low-dimensional real-valued vectors in which words that have similar meaning or are used in similar ways have a similar representation. Moreover, sequential deep learning models like recurrent neural networks (RNN) and long short-term memory networks lend themselves naturally to the task of processing sequences of free-flowing text, which enables them to capture the meaning of word-ordering for a variety of tasks like machine translation^35^ and named entity recognition.^36^

We have found that many of the most recent NLP application in pathology are designed based on deep learning models and use a distributed representation of words to represent the data input.^37^, ^38^, ^39^, ^40^, ^41^ John et al.^38^ investigated the usage of convolutional neural networks (CNN)^42^ to classify ICD codes from breast and lung cancer pathology reports. The authors reported their best system with a F1-micro score of 0.722 over 12 ICD-O-3 topography codes. Mohammed et al.^43^ proposed to use concept CUIs from UMLS as another source of information to a CNN model to improve model performance towards class imbalanced datasets.

The likely explanation for the improvement of performance of deep learning models over traditional statistical machine learning models is that these deep networks are able to learn very complex structures and relationships between words and labels that are often too complex to be observed by simpler techniques. Although DL methods have made many breakthroughs and have achieved state-of-the-art results in many clinical and non-clinical NLP tasks, these methods have several limitations. First, the number of trainable parameters is extremely high, making these methods very computationally expensive to train. As a consequence, DL models require a large volume of quality labeled data to be effectively trained and to be able to generalize on unseen data, also known as out-of-distribution data. There is also an issue of opacity - once a deep learning system has been trained, it is not always clear how the model makes decisions which limits the ability to troubleshoot in case of errors. As a result, deep learning models frequently lack interpretability, which can lead to unseen biases, decreased trust, and ultimately decreased adoption.

#### Hierarchical-deep Models

Despite recent advancements, adapting generic deep learning architectures to perform IE from pathology reports is challenging since pathology reports present a unique challenge where only a small fraction (usually 2–3 sentences) of a lengthy document is relevant to the specific classification task and positioning of that segment is not standard. Common DL models architectures like RNNs and CNNs have difficulty retaining information over long text sequences^44^ and they often cannot perform well under such specific conditions.

To overcome such limitations, recent work in pathology NLP has been focused on *attention* mechanisms^45^ and hierarchical models.^46^ Attention mechanisms learn relative importance of each textual token in the input text sequence in the context of downstream task such as classification, allowing them to focus on relevant parts of long input sequences. Hierarchical models leverage the hierarchical nature of textual input where words form sentences, and sentences form documents. Hence, word embeddings are aggregated to build sentence embeddings, and the model is trained to learn sequential sentence embedding to form document representation. The main goal of both approaches is to be able to get a more complex sentence and document representation, which can lead to a boost in performance of the deep learning model and also improve the interpretability of the models at multiple scales - sentence and word levels.^47^, ^48^, ^49^, ^50^, ^51^

Shang Gao et al.^49^ proposed a hierarchical self-attention networks (HiSANs) model for cancer pathology information extraction and text classification. Inspired by the transformer architecture^45^ and its attention mechanism, Shang Gao et al.^49^ proposed to replace the vanilla RNN part of the previous model (sentence-wise and word-wise) with a transformer self-attention layer. The authors reported a macro F1 score^52^ of 63.4, 50, 84, 30.2, and 74.3 for detecting site, laterality, behavior, histology, and tumor grade, respectively, using a large cancer pathology report dataset obtained from the National Cancer Institute’s Surveillance, Epidemiology, and End Results (SEER) program. To capture document-level relationships between clinical reports from the same patient, Shang Gao et al.^50^ expanded their previous HiSANs^49^ architecture to incorporate case-level context (aggregating information across multiple documents) from a sequence of related cancer pathology reports for information extraction and classification. The authors observed a substantial improvement in performance indicating that case-level context improved tumor site and morphology categorization considerably. Waheeda Saib et al.^51^ proposed a hierarchical CNN model to automate the classification of breast pathology reports into relevant International Classification of Disease for Oncology codes. The authors reported a micro and macro F1 of 0.918 and 0.692, respectively, for classifying 9 distinct ICD-O classes from 1813 anonymized unstructured breast cancer pathology reports. Although these methods have boost the performance of pathology report classification, they are often designed to operate on a single-task for extracting a particular characteristic. In consequence, different DL models must be developed for each task separately to generate a comprehensive understanding of pathology documentation (e.g., different cancer subgroups, stages), imposing additional duplicate workflows. Furthermore, the model does not consider possible shared key characteristics that might be common between different cancer types in pathology reports.

#### Multi-task learning (MTL)

Multi-task deep learning frameworks allow training of a unified model for several related downstream tasks such as part-of-speech tagging and named entity recognition from a text document.^53^ It is intuitive to assume that derived features useful for one predictive task must be relevant to another related predictive task. Multi-task frameworks allow related tasks to jointly learn and share hidden layer representations, improving quality of these representations and generalization capability of overall framework.^54^

Hong-Jun Yoon et al.^37^ were one of the first researchers to look into using MTL to extract information from pathology reports. They observed that MTL may be used in conjunction with DL models to enhance overall quality and performance when extracting information from pathology reports. Mohammed Alawad et al.^55^ proposed a single end-to-end multi-task CNN model to extract the primary site, histological grade, and laterality from unstructured cancer pathology text reports. In their work, they developed an approach using two stages. In the first stage, a MTL CNN model is trained for all tasks. Subsequently, using a transfer learning approach, the MTL CNN model parameters are used to initialize a CNN model for each individual task (one model per task). The drawback of their work is the limited number of cancer types and information extraction tasks performed. Another limitation was the size of the corpus, which included only a few hundred reports in each task. To address many of these challenges, Mohammed Alawad et al.^56^ improved their previous CNN network^55^ by adding a cross-stitch method to train a word-level CNN for the automatic extraction of cancer data from pathology reports. Their proposed method enhanced the state-of- the-art performance on IE from pathology reports by demonstrating how related information may be utilized to create shared representations. Although these methods have achieved state-of-the-art results on IE from cancer pathology reports, they are still very dependent on the CNN architecture and its limitations. For instance, because CNN models use a fixed window (which is usually small) for the convolution operation, these models can only capture linguistic relationships within that fixed window size of words. This brings back the challenge of how to extract relatively small elements of relevant information from lengthy pathology reports.

## 1 Discussion and Future Directions

Our systematic review shows there has been a considerable increase in the number of studies using NLP and deep learning on pathology reports in recent years. We observed that the majority of studies contain NLP systems that perform one of three tasks: (i) extraction of cancer characteristics (grade, stage, histology, laterality, etc.), (ii) classification of ICD 9/10 codes from cancer pathology reports, or (iii) extraction of anatomic information including lesion characteristics (size, shape, and cellular appearance of a specimen). The major benefit of using NLP is automation - such systems can reduce manual effort when extracting useful information in a large scale. In this section, we discuss the pros and cons of the recent publications and offer some insights of how NLP is developed and applied to the pathology domain. Furthermore, we discuss their limitations and offer some recommendations that summarize the recent trends.

### 1.1 Principal Findings

Recently, deep learning has been adapted for many NLP tasks and yielded promising results, including information extraction from pathology reports. The key difference between ML and DL approaches is the fact that unlike traditional ML models, DL algorithms do not use one-hot encoding for word representations. Instead, DL models represent words as embedded vectors, known as word embeddings. In deep learning, CNN architectures was the most popular method used among the reviewed papers. This recent trend is due to the fact that 1D-CNNs which are designed to find spatial patterns in images, are able to efficiently capture the semantics of short textual sequences from pathology reports and generate superior performance to ML models. Although ML and DL approaches are more powerful, they cannot be used for very small and/or imbalanced datasets, in which case rule-based/intelligent techniques may be more effective. Deep learning models also have an extremely high number of trainable parameters, making these methods very computationally expensive to train. One research direction to alleviate some of these limitations are the use of pre-trained models and shared-task representation. These approaches often only require fine-tuning the model for the downstream task, which reduces the computational cost. John et al.^38^ studied how word-vector representations using a CNN classifier performed consistently better than conventional Term Frequency-Inverse Document Frequency (TF-IDF)^58^ approaches when classifying ICD codes from pathology reports. Another method to improve DL model performance is to incorporate domain expertise knowledge. Mohammed et al.^43^ observed that the combination of word embeddings and CUIs from UMLS with a CNN classifier improved model performance towards class imbalanced on pathology datasets. Further work in this area could result in more stable and generalizable models and improve model performance when dealing with small or imbalanced datasets.

Lately, there has been a significant shift in NLP research toward bi-directional encoder representations from transformers (BERT).^59^ This algorithm utilizes a self-attention mechanism transformer,^45^ and is a context-preserving NLP technique. In other words, BERT can represent words or sequences in a way that captures the contextual information, causing the same sequence of words to have different representations when they appear in different contexts. In the clinical domain, specialized domain BERT models have been introduced such as BioBERT,^60^ ClinicalBERT,^61^ Blue-BERT,^62^ and CharacterBERT^63^ that are tailored for medical report interpretation. Shang Gao et al.^49^^,^^50^ proposed two hierarchical classification models where they replaced the vanilla RNN of the word and sentence representations with a transformer self-attention layer.^45^ They observed a substantial improvement of performance when compared with a traditional DL model like CNNs. They also found that these models can give a significant attention weight even for rare words, which are often present in underrepresented or undersampled entities. However, despite this new trend and the advancements to enhance text representation made by Transformer models,^45^ to our knowledge, there are no studies adapting BERT models to pathology tasks. Therefore, more exploration on the use of contextualized embeddings and attention mechanisms are needed in order to enhance pathology text representations.

Multiple pathology reports may be generated over the course of a patient’s disease and include information regarding evolution of the same malignancy. However, there are currently relatively few publications that look at how longitudinal data may be used to improve the overall quality and performance of cancer pathology report classification. It stands to reason that prior information is valuable for IE, given that a patient with a given cancer type is more likely to continue to have reports pertaining to the same cancer, hence narrowing the list of potential pathology entities to be classified in future reports. Mohammed Alawad et al.^56^ developed a hard parameter sharing word-level MT-CNN model and a cross-stitch word-level MT-CNN model that effectively showed how related information from multiple pathology reports could be used to learn shared representations across multiple tasks (primary site, laterality, grade, etc) and achieve state-of-the-art performance in classification accuracy. Future research should explore how these different aspects of a malignancy may be related and how these relationships can be incorporated with deep learning methods.

Finally, we observed that most studies remain in a proof-of-concept stage and have not yet been deployed in a clinical setting. We believe that there may be several reasons for the lack of implementation of NLP systems in routine clinical practice. First, although most studies used annotated data as their gold-standard for measuring model performance, inter-annotator agreement for most studies is unknown. In addition, some studies lack in describing the training, test, and validation splits. These measures are essential to ensure reproducibility and comparison to future work. Another issue is that the majority of the reviewed studies used retrospective data from a single institution when training and testing the NLP systems which may result in overfitting and decreased generalizability at external sites. Although performance of models should be assessed using multiple datasets across institutions, the availability of data remains limited due to ethics and privacy concerns.

### 1.2 Recommendations

This systematic review has shown that there is a clear necessity for pathology NLP systems to evolve beyond traditional deep learning methods. To progress the field, the following recommendations could be considered:

1. Increase focus on enhanced word and sentence representations. While progress has been made in using deep learning models and word embeddings for text representation, further efforts must be made to develop contextualized representations from pathology reports.
2. Focus on extracting multiple elements from pathology reports with shared model parameters and text embedding representations. The majority of pathology report datasets included in this review had many cancer types reported. As such, significant attention should be given to multi-task models to extract multiple cancer types and its characteristics.
3. Clarify methodologies. To enable inter-study comparisons and increase study reproducibility, authors must report study properties clearly, for example:
  • Data characteristics: dataset size, number of reports, sentences, unique words, distribution of the dataset, number of patients, demographic information of patients (if applicable), and others.
  • Performance metrics: authors should clearly state which metrics they are using. In addition, authors should not solely rely on just one evaluation metric, they should include a range of metrics, like precision, accuracy, recall, F1 score, receiver operating characteristic curve (ROC), area under the ROC curve (AUC), and many others.
  • Test of significance: When comparing different methods, a test of significance, like *p*-value test, should be performed.
4. Usage of prospective validation and external validation data. In order to avoid model over-fitting and to make generalizable models, authors should perform external validation. In addition, authors should also perform a prospective validation, where the most up-to-date data is reserved for testing.
5. In order to effectively advance pathology NLP systems, large-scale corpora must become available to researches. While other fields like radiology have shared datasets like MIMIC^64^ and i2b2,^65^ there are no publicly available pathology report datasets.

### 1.3 Limitations of Study

This systematic review examined the last 11 years literature of the use of NLP to automatic classify cancer pathology reports and may have the following limitations. We limited our search to only three online sources: PubMed, Science Direct, and Google Scholar. Furthermore, as the publication search is based on the reviewer criteria and the search keywords, it is subject to bias. Finally, the review is limited to articles written in English. While we try to be precise and objective during our review process, because of the listed limitations, it is possible that our search strategies did miss relevant publications.
